# Augustus Woodruff Brown

**Published:** 1895-07

**Authors:** 


### Obituary.

Augustus Woodruff Brown, D. D. S. Died July 5th at his Summer residence in Manchester, Vt., aged ninety. He was born in Litchfield, Conn., and was the oldest Dentist in America at the time of his death. He practiced in New York City for half a century, and retired with a fortune, fifteen years ago.

He was at first associated with Dr. Solyman Brown, his oldest brother, whose name is one of the best-known of early Dental literature. In their office at 13 Park Place, New York City, the first Dental Society in the world was organized and the first Dental Journal planned. These were the American Society of Dental Surgeons, of with Dr. Eleazer Parmly was first President and The American Journal of Dental Science, of which Dr. Solyman Brown was first Editor.

Dr. Augustus W. Brown at one time had the most aristocratic and lucrative practice in New York, and was widely known in social as well as professional circles.

One of the earliest Honorary Degrees of the Baltimore College of Dental Surgery was conferred upon him.

He married Miss Emma Mandeville, who survives him, together with two Daughters. He had nine children, but none of his sons survived to manhood.

Dr. E. Parmly Brown, son of his brother Solyman, studied with him and is the only member of the family now in Dentistry.

Dr. Samuel W. Stocton, uncle of Dr. S. S. White, and the original Manufacture of Teeth in America, married a cousin of Dr. A. W. Brown's wife, a Miss Seeley, whom he met at Dr. Brown's house.

The funeral services were held at Manchester, and the interment was in the family vault in old Marble Cemetry, New York City.

